# The effects of circuit hydraulic weight interval training on body composition and progression of resistance in recreationally exercising pre- and postmenopausal women: an 18-week quasi-experimental study

**DOI:** 10.3389/fphys.2025.1540983

**Published:** 2025-02-10

**Authors:** Małgorzata Socha, Paulina Ćwieląg, Waldemar Andrzejewski

**Affiliations:** ^1^ Department of Human Biology, Wroclaw University of Health and Sport Sciences, Wrocław, Poland; ^2^ Department of Fundamentals of Physiotherapy and Occupational Therapy, Wroclaw University of Health and Sport Sciences, Wrocław, Poland

**Keywords:** hydraulic resistance exercise, body composition, women, circuit training, interval training, body fat, muscle mass

## Abstract

**Background:**

The lack of physical activity, stress, and unhealthy eating habits contribute to body mass disorders, which form the basis of most civilization diseases. Mature women are increasingly turning to fitness clubs to improve their physique and protect themselves from diseases and the progressive aging process. The multitude of training systems proposed to women by fitness clubs leads to the search for solutions that will bring positive health results. The response to an exercise stimulus may depend on the menopausal status.

**Methods:**

This quasi-experimental study aimed to determine the effects of 6, 12, and 18 weeks of circuit hydraulic weight interval training (CHWIT) on anthropometric indices, body composition estimated by the bioelectrical impedance analysis (BIA), and muscle performance in inactive pre- and post-menopausal women from an urban population. A total of 100 women aged between 35 and 69 (mean 51.5 ± 9.61) years with a mean body mass index (BMI) of 27.3 (±5.4 kg/m^2^) were divided by menopausal status and assigned to the training CHWIT group (25 pre- and 25 postmenopausal women) and the control group (25 pre- and 25 postmenopausal women). Each participant from the CHWIT group took part in a total of 54 training sessions, developed for the Mrs.Sporty network, under the constant supervision of a qualified trainer.

**Results:**

After 18 weeks of training in both intervention groups, ANCOVA demonstrated statistically significant (*p* < 0.05) decreased body fat (%), reduced thigh and arm circumference, and increased muscle component (kg) as the main part of fat-free body mass. Additionally, premenopausal women decreased their body mass, BMI, and waist and hip circumferences. A significant increase in the muscle component was noticed after 6 weeks of CHWIT in pre-menopausal women and only after 18 weeks in postmenopausal women. Significant progression of resistance (amount of repetitions on hydraulic machines) was observed after 6 weeks and at each subsequent stage of CHWIT in both intervention groups (*p* < 0.001). No significant differences were found in the controls.

**Conclusion:**

CHWIT is an effective form of training, improving body composition and physical functions in inactive pre- and postmenopausal women. Changes in the muscle component require a longer intervention of physical effort in women after menopause.

## 1 Introduction

In the era of promoting physical activity, one of the ways of spending free time, especially in big cities, is working out in fitness clubs. Commercial sporting club networks play an important role in increasing the availability of exercise programs tailored to specific customer groups. Due to a growing population of older people, a greater interest in physical activity is observed among persons aged 35–54 years or even 55 and older, which are the groups with the highest percentage of physical inactivity (Physical Activity Council, 2019). It is estimated that approximately 75% of the recipients of fitness club services are mature women between the ages of 30 and 60 years who are interested in physical activity as a form of improving their body shape and protection against diseases and progression of the aging process (Physical Activity Council, 2019; Kerksick et al., 2009; de Mendonca et al., 2014). Sedentary lifestyle, stress, or unhealthy diets contribute to body mass disorders, which form the basis for the majority of civilization diseases. Many physiological changes that accompany women at the onset of menopause enhance the activity of risk factors related to improper lifestyle. Menopause is associated with a natural decrease in estrogen, which enlarges visceral fat mass, diminishes bone mass density, and reduces the strength and mass of skeletal muscles, contributing to a higher risk of falls and fractures (Maltais et al., 2009; Buch et al., 2016). It has been indicated in many controlled, clinical studies that the most effective method of building muscle mass in adult women is weight training (WT). Developing the strength and size of muscles after WT is a consequence of muscle satellite cell recruitment initiated in order to support adult muscle fiber hypertrophy (Hunter et al., 2004). Other beneficial effects of WT include the improvement of bone density and insulin sensitivity and the decrease in the risk of being overweight and the incidence of obesity-related diseases (Strasser and Schobersberger, 2011; Csapo and Alegre, 2016; Socha et al., 2016). Recent research (Nilsson et al., 2023) has shown that the implementation of a 15-week WT regimen in postmenopausal women can help counteract the abdominal fat redistribution associated with the menopausal transition. In WT, the individual’s own weight, free weights, or machines may be used. A friendly, as well as more willingly chosen, alternative for women, especially in the menopausal period, includes hydraulic machines for WT. These react with adjustable resistance determined by the amount of effort applied by the exercising person (Takeshima et al., 2002; Falcone et al., 2015).

WT with the hydraulic exercise equipment (HWT) minimizes pain and muscle fatigue along with the hazard of injuries related to eccentric contractions (Gabriel, 1991; Lee et al., 2011). Due to these reasons, it is recommended for older people (Lee et al., 2011), children (Weltman et al., 1986), and sick persons who may have contraindications to classic resistance exercise (Suomi et al., 1995; Ha et al., 2015). The research on these groups indicated that a low level of resistance, dependent on the effort and speed used by an individual at each repetition, may be enough and bring expected benefits of improved strength and muscle mass (Lee et al., 2011). Meta-analysis of WT influence on muscle mass in older persons demonstrated that with enough amount of repetitions, lower weights than those that are traditionally advised were enough to evoke considerable growth of muscle strength (Csapo and Alegre, 2016).

Hydraulic exercise equipment may be used for circuit training (CHWT) while alternating resistance with endurance and coordination workout. Such form of training improves body composition, muscle strength, and the functioning of the cardiovascular system and, as a consequence, helps maintain functional efficiency during aging (Romero-Arenas et al., 2013; Morgan et al., 2023). The comparison of energy expenditures between circuit training (CT) and traditional resistance exercises showed higher energy expenditure during circuit training (Pichon et al., 1996). Exercise protocols that provide differentiated intensity of practice are recommended for individuals who are older, overweight, and have obesity or co-existing diseases (Klika and Jordan, 2013; Marcos-Pardo et al., 2019).

Interval training (IT) is such a form of exercise, which includes alternating periods of work and rest during a training session. This kind of practice improves the functioning of the cardiovascular system and increases lipid utilization as an energy substrate (Schoenfeld and Dawes, 2009), and it may be a part of CT protocol (Kravitz, 1996). WT is a method recommended by fitness specialists in many systems of workout such as circuit weight training (CWT), interval weight training (IWT), and circuit weight interval training (CWIT). It was proven that the combination of CWIT in professionally active women aged 34.0 (±5.3) years provides better physiological benefits (muscular endurance, strength, and cardiovascular endurance) than traditional CT methods (Skidmore et al., 2012).

As for health reasons, the post-training modifications of body composition are a matter of importance. These include reduction of body fat and, due to the involution processes in muscle tissue, changes in the proportions of body fat and fat-free mass. Combining CWT and IT increases energy expenditure and is an option recommended for people expecting maximum fitness results in a short period of time (Skidmore et al., 2012). Recent reviews provide evidence that short, intense, intermittent exercise interventions included in HIIT (high-intensity interval training) programs induce beneficial changes in anthropometric indices, body composition, and cardiometabolic and mental health parameters in overweight and obese adult populations (Batrakoulis and Fatouros, 2022; Batrakoulis et al., 2021a; Batrakoulis et al., 2022). Fitness programs for older adults, exercise for weight loss, traditional strength training, HIIT, exercise for mental health, and functional fitness training were among the top 10 fitness trends for 2025 (Newsome et al., 2024). The authors of this study emphasize the role of these programs in maintaining overall health, disease prevention, and independent living in a cohort of overweight older adults. Batrakoulis et al. (2021a) suggested a higher efficacy for multi-component exercise interventions (hybrid training) compared to single-component modalities.

In view of the above, we assumed that the circuit hydraulic weight interval training (CHWIT) with variable intensities joined with short periods of active rest, which was developed by Mrs.Sporty, will be a proper form of workout for peri-menopausal women training recreationally. There are no comparative data on the influence of CHWIT on the body composition of women in the pre- and postmenopausal periods, especially those indicating changes in the muscle component of the body. This study analyzed the effects of a 6-, 12-, and 18-week-long CHWIT program, which combined hydraulic resistance with circuit and interval training, on anthropometric indices, body composition, and muscle performance in pre- and postmenopausal women in order to clarify whether the role of CHWIT is also applicable in losing body fat and increasing the muscle component of the body in comparable age groups.

## 2 Materials and methods

### 2.1 Participants

**FIGURE 1:**
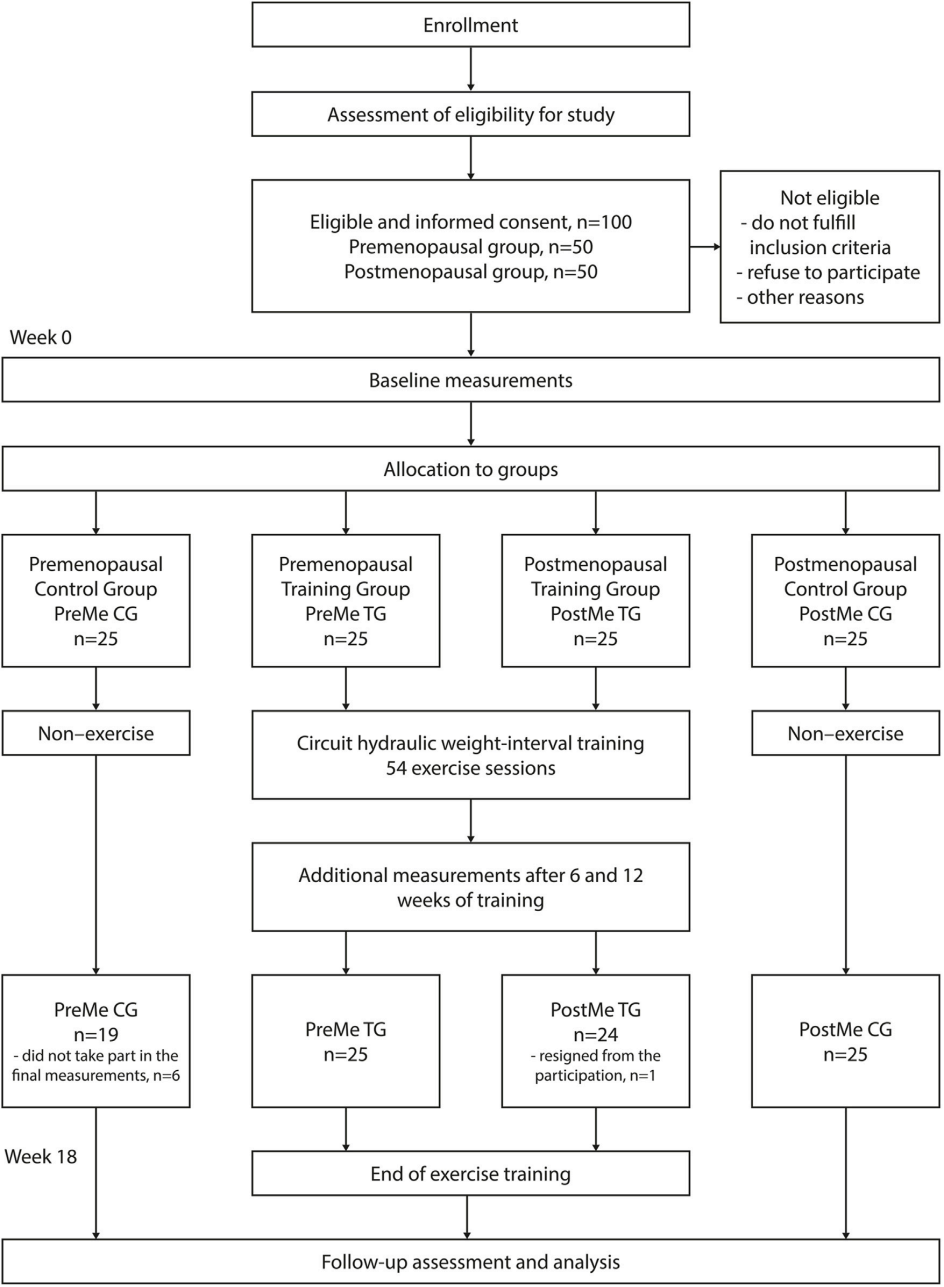
Flowchart of participant selection and the study process.

This quasi-experimental 18-week-long study involved collaboration with the Mrs.Sporty commercial sports club network (mrssporty.pl). We received approval to cooperate with a local recreational sports center for women, Mrs.Sporty in Wrocław (Poland). Participants recruited for the project were women aged between 35–69 years from an urban population, who had not taken part in any type of sports training for at least a year and had declared their regular involvement in the recreational training sessions. Study participants had to have permission from their family doctor to take up the exercise. Individuals with any physical and/or psychological conditions or those taking any medications were not included in the study. The flowchart of this study according to the Consolidated Standards of Reporting Trials (CONSORT) is depicted in Figure 1. The sample size was estimated *a priori* based on a repeated-measures ANOVA test, with an interaction between the two groups, using G*Power software (version 3.1.9.7) (Beck, 2013). For an effect size of 0.25, power of 0.90, and with an alpha level of p ≤ 0.05, the required total sample size for this study was 30 subjects.

Peri-menopausal women were enrolled on the basis of a sports club invitation. At recruitment, the subjects were divided into pre- and postmenopausal groups. The women were regarded as post-menopausal if they had stopped menstruating at least a year before the study started or if they were aged ≥55 years. Finally, the project enlisted 50 premenopausal women aged between 38 and 49 (44.1 ± 3.08) years and 50 postmenopausal women aged between 50 and 69 (59.9 ± 5.83) years with body height 165.9 (±5.1) cm and 162.7 (±5.2) cm, respectively (p = 0.003). After enrolling for the study, the participants of both the pre- and postmenopausal groups were assigned to either the CHWIT or control groups. The participants declared that they would not change their diet throughout the study period of 18 weeks or attend any other physical activity exceeding their regular, daily level of activity. Before the first exercise session, a training session regarding the appropriate position on each hydraulic machine and the proper manner of exercise was organized. During each subsequent session, a personal trainer supervised the exercise and corrected the body position.

All subjects provided written and informed consent to take part in the study in accordance with the Declaration of Helsinki. The research project was approved by the local Research Ethics Committee of the Wroclaw University of Health and Sport Sciences (No. 272014).

### 2.2 Measurements

BIA, as well as dual-energy X-ray absorptiometry (DXA), ultrasonography, and anthropometry, are commonly used as secondary, indirect methods to assess human body composition, including skeletal muscle mass as a fat-free body mass component (Yamada, 2018; Gobbo and Cyrino, 2021). As a recent review has shown, each of the methods mentioned above has limitations, and they all allow the estimation of body composition based on several assumptions (Holmes and Racette, 2021). In our study, we used anthropometric and BIA methods. The choice of body composition assessment method was determined by the availability, cost, group size, location of the study, and the need to repeat measurements every 6 weeks. Each participant had their body height (cm); body mass (kg); and the circumferences of the waist (cm), hips (cm), right thigh (cm), and right upper arm (cm) measured. The indexes of BMI (kg/m^2^) and WHR (waist–hip ratio) were calculated. Body fat (%) and muscle component (kg) as elements of body composition were estimated, applying a BIA using a TANITA BC- 418 MA analyzer (Japan) with GMON software version 3.1.8. The assessment of the validity and reliability of body composition estimation with this analyzer was carried out by Kelly and Metcalfe (2012). Because BIA is extremely sensitive to changes in body hydration, we have taken steps to minimize the estimation error. In order to eliminate any circumstances affecting fluid imbalance in the body, study participants were given thorough instructions regarding hydration, exercise, sleep, diet, alcohol consumption, the use of diuretics, and the presence of edema (Dżygadło et al., 2012). Body composition tests were performed in the morning. In addition, the subjects were to empty their bladder 30 min before the test. If the color of the urine was dark, they were to drink water and wait for 45 min before being assessed (Holmes and Racette, 2021).

Muscle performance was determined by the maximum number of repetitions of an exercise on each of eight hydraulic machines used in the study. In both the experimental age groups, the measurements were carried out before the series of exercise and then after 6, 12, and 18 weeks of training. At each stage of the study, the effects of exercises were discussed with the participants and another training aim was established. In the control groups, the measurement were carried out before and after the intervention, i.e., in the first and 18th week of the program.

### 2.3 Training protocols

The training offered by the Mrs.Sporty sports center in Wrocław lasted 18 weeks, and the subjects performed three CHWIT workouts per week. Each participant took part in a total of 54 training sessions during the study. Exercises were conducted in two age groups, namely, pre- and postmenopausal groups, under the constant supervision of a qualified trainer. The CHWIT training protocol consisted of three full workout circuits on the hydraulic gym machines based on controlled resistance, which addressed the main muscle groups. The pressure ranges on each machine was set a level of three bars. The exercises were performed in a continuous manner, at a maximum effort on the machines, with repeated intervals of 40 s, between which there were 40-second-long periods of active rest on the transition stations. The participants were instructed to perform as many repetitions as possible on every hydraulic machine. A single training session lasted approximately 32–34 min, with additional time for stretching (eight stretching exercises; each position for a minimum of 30 s). One circuit consisted of eight stations of hydraulic machines and the same number of transition stations set alternately. The training began with exercise at the active rest station and finished on the last machine. After each circuit, there was a heart rate measurement lasting 10 s. One half of the first circuit was treated as a warm-up, and the work done was at the level of approximately 50% of the maximum heart rate. Then, after the fourth machine, acceleration took place, and at this stage, the work was at 60%–70% of the maximum heart rate. Such a pace was maintained until the second half of the third circuit, during which the intensity of exercise and heart rate decreased. After completion of the last circuit, there was a standard procedure of whole-body stretching.

In the study, the following machines with hydraulic resistance were used: pulldown/shoulder press (strengthening the muscles of shoulders, chest, neck, upper back, and arms), adductor/abductor (strengthens inner and outer thigh, as well as hip muscles), chest press/rowing (training muscles of chest, shoulders, neck, upper back, and arms), hip extension 1 and hip extension 2 (build up gluteal muscles and flexors of lower limbs), back extension/flexion (training abdominal and back muscles), butterfly/butterfly reverse (strengthening chest and back muscles), and squat (strengthens lower limbs’ muscles). At the transition stations, the participants exercised with equipment listed below: fitness ball (lifting the ball over head and raising knees seated on the ball), thera-bands (bending and straightening movements of the elbow and shoulder abduction movements), Bosu balls (squats, balancing on the ball, and stepping on and off the ball), gymnastic rods (lifting and twists), flexibars (vibrating in front of the chest both in vertical and horizontal positions, in one upper limb, and over the head), small exercise balls (squeezing in front of the chest and between thighs and pressing a foot into the ball), platforms (squats, forward step-ups, and shifting a small ball under the knees), and aerobic steps (stepping up and down with weights (1.0, 1.5, and 2.0 kg) kept in upper limbs, joining right elbow with the left knee and vice-versa, three knee raises, and leg-kicking).

### 2.4 Statistical analysis

In order to obtain results, data analysis was carried out using the Statistica software package (version 13.3, license from StatSoft Polska, Kraków, Poland). Results are expressed as mean ± standard deviation or mean changes and 95% confidence intervals (CIs). Normality of the distribution (Kolmogorov–Smirnov test) and homogeneity of variance (Levene’s test) were determined for all outcome measures. Sample characteristics at baseline were compared between groups using an independent-means Student’s t-test, and additionally, paired samples *t*-tests were used to examine effects by time (before to after intervention). As the effects of the training were assessed before training and at three time points (after 6, 12, and 18 weeks of the exercise program), to assess the within-group differences, a repeated-measures analysis of variance (ANOVA) was carried out. Primary statistical analysis was the determination of between-group differences using one-way analysis of covariance (ANCOVA) to evaluate the main and intervention effects. ANCOVA was used to evaluate variables that were measured at only two time points using the 18-week value as the dependent variable and the baseline value as covariate. The *post hoc* Bonferroni test was used for both analyses when a considerable difference between the samples was revealed. Variation in each variable based on time was calculated as the post intervention value – pre intervention value. All statistical tests were performed considering a significance level of *p* < 0.05. To assess the magnitude of differences between groups, the effect size (ES) was calculated in relation to the statistical tests used in the research plan (Cohen’s d for t-tests and partial eta squared for ANOVA and ANCOVA). Based on Cohen (1988), we considered an ES of 0.20–0.49 to be small, 0.50–0.79 as moderate, and ≥0.80 as high.

## 3 Results

The project was completed by 93 women; in the training groups, one of the women resigned from participation in the program during the third week of practice, whilst six persons from the control groups did not take part in the final measurements. In the result, 49 women accomplished 54 CHWIT sessions after 18 weeks and were considered for the final analysis (Figure 1). Over the entire 18-week intervention period, the mean adherence rate was 92.6%, with adherence rates ranging from 80% to 100%. No adverse events were also reported during the intervention period. Inter- and intra-group differences in pre–post training assessment of anthropometric and body composition characteristics are presented in Table 1 for premenopausal women and in Table 2 for post-menopausal women. At the starting point, there were no differences between the control and intervention groups and between pre- and postmenopausal groups with the reference to body mass, muscle component, trunk and limbs’ circumferences, BMI, and WHR indexes. There were also no statistically significant differences in fat percentage between the intervention and control groups in both age groups. However, the compared pre- and postmenopausal groups varied significantly in the level of body fat, which was approximately 5% higher in women after menopause (ES = 0.66; *p* = 0.013).

**TABLE 1:** Effects of CHWIT on anthropometric and body composition indices in premenopausal women.

Variable	Group	Baseline	6 weeks	12 weeks	18 weeks	F	*p* value^c^	Difference in change	95% CI for change	F	*p*-value^d^
Weight (kg)	CHWIT control	73.0 ± 17.0 72.8 ± 17.4	72.4 ± 16.9 *^,a^	71.6 ± 16.6 *^a,b^	71.3 ± 16.3 *^,a^ 73.9 ± 17.3 *^,a^	9.2	<0.001	−2.6	−3.6 to −1.6	31.8	<0.001
BMI (kg/m^2^)	CHWIT control	26.6 ± 5.7 26.1 ± 5.8	26.4 ± 5.6 *^,a^	26.1 ± 5.5 *^,a,b^	26.0 ± 5.4 *^,a^ 26.5 ± 5.7 *^a^	9.8	<0.001	−0.5	−0.9 to −0.2	32.3	0.005
Body fat (%)	CHWIT control	34.0 ± 8.9 32.9 ± 8.5	33.3 ± 0.9 *^,a^	32.7 ± 8.7 *^,a,b^	32.2 ± 8.6 *^a,b^ 34.1 ± 8.8 *^,a^	26.6	<0.001	−3.4	−4.5 to −2.3	14.7	<0.001
Muscle component (kg)	CHWIT control	44.4 ± 5.3 45.2 ± 5.4	44.8 ± 5.6 *^,a^	44.8 ± 5.7 *^,a^	45.3 ± 5.8 *^,a,b^ 44.3^a^ ± 5.1	15.5	<0.001	1.5	0.9 to 2.1	33.9	<0.001
Waist (cm)	CHWIT control	87.9 ± 14.5 82.4 ± 14.2	85.9 ± 14.1*^,a^	85.0 ± 13.9 *^,a,b^	84.0 ± 13.8 *^,a,b^ 82.9 ± 14.2^a^	27.5	<0.001	−4.0	−5.5 to −2.4	14.3	<0.001
Hips (cm)	CHWIT control	104.6 ± 11.0 101.0 ± 14.0	103.4 ± 10.9 *^,a^	102.6 ± 10.9 *^,a,b^	101.7 ± 10.8 *^,a,b^ 101.4 ± 14.1 *^,a^	36.9	<0.001	−4.9	−6.0 to −3.8	20.8	<0.001
WHR	CHWIT control	0.84 ± 0.07 0.82 ± 0.01	0.83 ± 0.07 *^,a^	0.82 ± 0.07 *^,a^	0.82 ± 0.07 *^,a^ 0.82 ± 0.06	4.6	0.005	−0.01	−0.02 to 0.01	4.0	0.408
Right thigh (cm)	CHWIT control	59.4 ± 7.3 58.9 ± 7.5	58.5 ± 7.1 *^,a^	58.1 ± 6.8 *^,a,b^	57.8 ± 6.7 *^,a,b^ 59.2 ± 7.4	16.1	<0.001	−1.5	−2.1 to −0.9	39.3	<0.001
Right arm (cm)	CHWIT control	30.8 ± 4.2 30.3 ± 5.0	30.5 ± 4.3 *^,a^	30.2 ± 4.1*^,a,b^	30.0 ± 4.0 *^,a,b^ 30.7 ± 4.8	11.3	<0.001	−0.6	−1.0 to −0.2	25.9	0.006

The values were presented as mean ± standard deviation, mean difference in the change (post intervention value – pre intervention value), and 95% confidence interval (CI) for differences; CHWIT, circuit hydraulic weight interval training)-experimental group (n = 25); control, control group (n = 19); BMI, body mass index; WHR, waist to hip ratio; * statistically significant; ^a^significant difference from baseline, *p* < 0.05; ^b^significant difference from last measurement, *p* < 0.05; ^c^calculated by multivariate tests for repeated measures ANOVA; ^d^comparing between CHWIT and control group calculated by ANCOVA; analysis adjusted for baseline values.

**TABLE 2:** Effects of CHWIT on anthropometric and body composition indices in postmenopausal women.

Variable	Group	Baseline	6 weeks	12 weeks	18 weeks	F	*p*-value^c^	Difference in change	95% CI for change	F	*p*-value^d^
Weight (kg)	CHWIT control	74.4 ± 15.1 73.0 ± 10.8	73.5 ± 14.5 *^,a^	72.9 ± 14.2 *^,a,b^	72.4 ± 13.9 *^,a,b^ 73.5 ± 10.4	15.3	<0.001	−0.2	−0.8 to 0.5	45.7	0.635
BMI (kg/m^2^)	CHWIT control	28.0 ± 5.1 27.5 ± 4.1	27.7 ± 4.9 *^,a^	27.4 ± 4.8 *^,a,b^	27.3 ± 4.7 *^,a,b^ 27.7 ± 4.0^a^	17.4	<0.001	0.2	−0.1 to 0.4	38.6	0.223
Body fat (%)	CHWIT control	39.1 ± 7.7 36.7 ± 5.1	38.4 ± 7.9 *^,a^	37.9 ± 7.8 *^,a,b^	37.3 ± 7.6 *^,a,b^ 37.8 ± 5.0^a^	26.6	<0.001	−1.9	−2.7 to 1.0	23.4	<0.001
Muscle component (kg)	CHWIT control	43.2 ± 4.6 43.5 ± 3.5	43.2 ± 4.7	43.4 ± 4.7	43.7 ± 4.9 *^,a,b^ 42.9 ± 3.3 *^,a^	5.1	0.003	1.1	0.6 to 1.5	23.0	<0.001
Waist (cm)	CHWIT control	92.5 ± 12.9 89.0 ± 13.9	90.4 ± 12.6 *^,a^	89.1 ± 12.2 *^,a,b^	87.8 ± 11.8 *^,a,b^ 89.6 ± 14.1^a^	55.4	<0.001	0.3	−0.8 to 1.3	92.1	0.619
Hips (cm)	CHWIT control	106.6 ± 9.6 104.7 ± 10.3	105.2 ± 9.0 *^,a^	104.4 ± 9.0 *^,a,b^	103.6 ± 8.7 *^,a,b^ 105.1 ± 10.5 *^,a^	63.4	<0.001	−0.2	−1.3 to 0.9	11.4	0.695
WHR	CHWIT control	0.87 ± 0.07 0.85 ± 0.07	0.86 ± 0.07 *^,a^	0.85 ± 0.06 *^,a,b^	0.84 ± 0.06 *^,a,b^ 0.85 ± 0.06	7.1	0.002	0.01	−0.004 to 0.02	3.9	0.161
Right thigh (cm)	CHWIT control	59.1 ± 5.5 57.7 ± 4.4	58.2 ± 5.3 *^,a^	57.7 ± 5.2 *^,a,b^	57.5 ± 5.2 *^,a,b^ 57.8 ± 4.3	34.5	<0.001	−0.6	−1.0 to 0.1	16.7	0.01
Right arm (cm)	CHWIT control	32.0 ± 3.9 31.0 ± 2.7	31.5 ± 3.9 *^,a^	30.9 ± 3.8 *^,a,b^	30.7 ± 3.6 *^,a,b^ 31.2 ± 2.9	19.0	<0.001	−0.5	−0.9 to 0.0	42.7	0.04

The values were presented as mean ± standard deviation, mean difference in the change (post intervention value – pre intervention value), and 95% confidence interval (CI) for differences; CHWIT, circuit hydraulic weight-interval training-experimental group (n = 25); control, control group (n = 19); BMI, body mass index; WHR, waist to hip ratio; * statistically significant; ^a^significant difference from baseline, *p* < 0.05; ^b^significant difference from last measurement, *p* < 0.05; ^c^calculated by multivariate tests for repeated measures ANOVA; ^d^comparing between CHWIT and control group calculated by ANCOVA; analysis adjusted for baseline values.

After 6 weeks of training, both the experimental pre- and post-menopausal groups showed statistically significant differences (p < 0.05) in body weight, BMI, body fat (%), trunk and limb circumferences, and WHR index (Tables 1, 2). After 18 weeks of training, multivariate tests for repeated-measures ANOVA showed statistically significant decreases in body weight (PreMe: −1.7 ± 1.9 kg, ES = 0.39, p < 0.001; PostMe: −2.0 ± 2.0 kg, ES = 0.48, p < 0.001); BMI (PreMe: −0.6 ± 0.7 kg/m^2^, ES = 0.40, p < 0.001; PostMe: −0.7 ± 0.8 kg/m^2^, ES = 0.48, p < 0.001); body fat (PreMe: 1.7% ± 1.4%, ES = 0.53, p < 0.001; PostMe: −1.8% ± 1.4%, ES = 0.51, p < 0.001); waist (PreMe: −3.9 ± 2.5 cm, ES = 0.68, p < 0.001; PostMe: −4.7 ± 2.3 cm, ES = 0.75, p < 0.001), hips (PreMe: −2.9 ± 1.7 cm, ES = 0.71, p < 0.001; PostMe: −3.0 ± 2.2 cm, ES = 0.59, p < 0.001), thigh (PreMe: −1.6 ± 1.2 cm, ES = 0.59, p < 0.001; PostMe: −1.7 ± 1.0 cm, ES = 0.66, p < 0.001), and arm (PreMe: 0.8 ± 0.8 cm, ES = 0.46, p < 0.001; PostMe: −1.3 ± 1.0 cm, ES = 0.61, p < 0.001) circumference; and WHR (PreMe: −0.01 ± 0.02, ES = 0.28, p = 0.005; PostMe: −0.03 ± 0.02, ES = 0.41, p = 0.002) (Tables 1, 2). The main difference between pre- and postmenopausal group in response to prolonged physical activity was associated with changes in the muscle component (kg) as the main part of fat-free body mass. In pre-menopausal women, a significant increase in the muscle component estimated by BIA was noted after 6 weeks of CHWIT (+0.4 ± 0.6 kg; ES = 0.60; *p* = 0.005) and after 18 weeks of training (+0.9 ± 1.0 kg; ES = 0.31; *p* < 0.001), while in the group of postmenopausal women, it was seen only after 18 weeks of training (+0.5 ± 0.9 kg; ES = 0.18; *p* = 0.003).

In the group of premenopausal women, ANCOVA (Table1) verified the null hypothesis that the mean values in the two groups are not different after 18 weeks CHWIT accounting for baseline values. After training, compared to the control, the intervention group showed significant differences for each anthropometric and body composition variable, except for the WHR index (*p* = 0.408). Body mass (ES = 0.41, *p* < 0.001); BMI (ES = 0.42, *p* = 0.005); body fat (%) (ES = 0.57, *p* < 0.001); and waist (ES = 0.57 *p* < 0.001), hip (ES = 0.62, *p* < 0.001), thigh (ES = 0.51, *p* < 0.001), and arm (ES = 0.38, *p* = 0.006) circumferences were decreased after training in the CHWIT premenopausal group. Muscle component (kg) was statistically significantly increased after 18 weeks of CHWIT in premenopausal group (ES = 0.43, *p* < 0.001). In postmenopausal women (Table 2), ANCOVA revealed significant differences between groups post 18 weeks CHWIT only in the case of body fat (%) (ES = 0.60; *p* < 0.001), muscle component (kg) (ES = 0.36, *p* < 0.001), and thigh (ES = 0.58, *p* < 0.001) and arm circumferences (ES = 0.50, *p* < 0.04), and there was no significant between-group differences for the remaining outcomes (*p* < 0.05). The values of these variables, except the muscle component, decreased in the intervention group.

Regarding resistance progression, measured by the number of repetitions on the hydraulic machines in subsequent weeks of CHWIT, cross-group comparisons are presented in Table 3 for premenopausal women and in Table 4 for postmenopausal women. At the beginning of the research, there were no statistically significant differences in resistance between the intervention and control groups in both age ranges. Pre-menopausal women performed a significantly higher amount of repetitions than postmenopausal women only on the hip extension machines, both at the beginning of the study and after 18 weeks of training (ES = 0.36–0.76, *p* = 0.02–0.04), which proves a greater strength of gluteal muscles and lower limbs’ flexors. In both age groups, increases in resistance occurred progressively and were identified within the first 6 weeks of the intervention, and each subsequent 6 weeks of CHWIT resulted in a significant increase in the number of repetitions performed on every hydraulic machine (p < 0.05). After 18 weeks of training, multivariate tests for repeated measures ANOVA showed statistically significant increases in the number of repetitions in pulldown/shoulder press (PreMe: +7.2 ± 3.8, ES = 0.66, *p* < 0.001; PostMe: +7.3 ± 3.1, ES = 0.74, *p* < 0.001), adductor/abductor (PreMe: +10.1 ± 4.1, ES = 0.71, *p* < 0.001; PostMe: +10.3 ± 5.3, ES = 0.69, *p* < 0.001), chest press/rowing (PreMe: +10.2 ± 4.7, ES = 0.73, *p* < 0.001; PostMe: +8.7 ± 3.8, ES = 0.77, *p* < 0.001), hip extension 1 (PreMe: +12.1 ± 4.5, ES = 0.74, *p* < 0.001; PostMe: +10.9 ± 4.6, ES = 0.77, *p* < 0.001), hip extension 2 (PreMe: +11.8 ± 5.7, ES = 0.72, *p* < 0.001; PostMe: +11.2 ± 4.2, ES = 0.76, *p* < 0.001), back extension/flexion (PreMe: +6.4 ± 3.5, ES = 0.69, *p* < 0.001; PostMe: +6.5 ± 3.3, ES = 0.67, *p* < 0.001), butterfly (PreMe: +8.2 ± 3.6, ES = 0.69, *p* < 0.001; PostMe: +9.1 ± 3.8, ES = 0.76, *p* < 0.001), and squat (PreMe: +9.0 ± 5.2,ES = 0.68, *p* < 0.001; PostMe: +8.0 ± 5.0, ES = 0.66, *p* < 0.001). These results have been confirmed by ANCOVA, which revealed significant between-groups effects: pulldown/shoulder press (PreMe: ES = 0.50, *p* < 0.001; PostMe: ES = 0.61, *p* < 0.001), adductor/abductor (PreMe: ES = 0.62, *p* < 0.001; PostMe: ES = 0.61, *p* < 0.001), chest press/rowing (PreMe: ES = 0.56, *p* < 0.001; PostMe: ES = 0.49, *p* < 0.001), hip extension 1 (PreMe: ES = 0.68, *p* < 0.001; PostMe: ES = 0.56, *p* < 0.001), hip extension 2 (PreMe: ES = 0.69, *p* < 0.001; PostMe: ES = 0.58, *p* < 0.001), back extension/flexion (PreMe: ES = 0.51, *p* < 0.001; PostMe: ES = 0.53, *p* < 0.001), butterfly (PreMe: ES = 0.65, *p* < 0.001; PostMe: ES = 0.69, *p* < 0.001), and squat (PreMe: ES = 0.60, *p* < 0.001; PostMe: ES = 0.40, *p* < 0.001). No statistically significant change in muscle performance was found in the control groups during the 18-week follow-up period (Tables 3, 4).

**TABLE 3:** Progression of resistance measured by the number of repetitions on hydraulic machines in premenopausal women.

Hydraulic machine	Group	Baseline	6 weeks	12 weeks	18 weeks	F	*p*-value^c^	Difference in change	95% CI for change	F	*p*-value^d^
Pulldown/shoulder press	CHWIT control	25.0 ± 4.7 25.4 ± 3.6	28.3 ± 4.5 *^,a^	30.0 ± 4.2 *^,a,b^	32.2 ± 3.8 *^,a,b^ 25.8 ± 5.0	29.3	<0.001	6.8	4.7 to 9.0	45.3	<0.001
Adductor/abductor	CHWIT control	33.2 ± 5.4 32.5 ± 4.9	37.9 ± 5.1 *^,a^	41.5 ± 5.1 *^,a,b^	43.3 ± 5.0 *^,a,b^ 32.6 ± 6.1	49.8	<0.001	11.7	9.2 to 14.2	69.4	<0.001
Chest press/rowing	CHWIT control	23.9 ± 5.6 23.8 ± 5.0	28.1 ± 4.8 *^,a^	31.8 ± 5.6 *^,a,b^	34.1 ± 5.3 *^,a,b^ 23.0 ± 5.7	41.0	<0.001	9.7	7.1 to 12.3	56.7	<0.001
Hip extension 1	CHWIT control	29.5 ± 5.0 27.7 ± 4.7	34.3 ± 5.2 *^,a^	38.7 ± 6.5 *^,a,b^	41.7 ± 4.9 *^,a,b^ 26.8 ± 5.3	64.6	<0.001	13.3	10.8 to 15.8	93.7	<0.001
Hip extension 2	CHWIT control	29.6 ± 5.5 27.5 ± 4.5	34.2 ± 4.9 *^,a^	38.5 ± 5.5 *^,a,b^	41.3 ± 4.4 *^,a,b^ 27.7 ± 5.4	38.8	<0.001	14.2	11.5 to 16.8	98.7	<0.001
Back extension/flexion	CHWIT control	15.3 ± 3.7 15.5 ± 3.8	18.3 ± 3.7 *^,a^	19.6 ± 4.2 *^,a,b^	21.7 ± 4.1 *^,a,b^ 15.8 ± 4.7	30.1	<0.001	6.3	4.4 to 8.3	45.2	<0.001
Butterfly	CHWIT control	22.3 ± 4.7 21.3 ± 4.5	25.7 ± 3.9 *^,a^	28.2 ± 4.4 *^,a,b^	30.5 ± 4.3 *^,a,b^ 22.0 ± 5.1	49.3	<0.001	10.0	7.9 to 12.0	81.3	<0.001
Squat	CHWIT control	23.3 ± 6.2 22.8 ± 5.6	27.4 ± 5.6 *^,a^	30.0 ± 4.9 *^,a,b^	32.3 ± 4.7 *^,a,b^ 21.9 ± 4.4	24.5	<0.001	9.7	7.3 to 12.0	66.5	<0.001

The values were presented as mean ± standard deviation, mean difference in the change (post intervention value – pre intervention value), and 95% confidence interval (CI) for differences; CHWIT, circuit hydraulic weight-interval training-experimental group (n = 25); control, control group (n = 19); * statistically significant; ^a^significant difference from baseline, *p* < 0.05; ^b^significant difference from last measurement, *p* < 0.05; ^c^calculated by multivariate tests for repeated measures ANOVA; ^d^comparing between CHWIT and control group calculated by ANCOVA; analysis adjusted for baseline values.

**TABLE 4:** Progression of resistance measured by the number of repetitions on hydraulic machines in postmenopausal women.

Hydraulic machine	Group	Baseline	6 weeks	12 weeks	18 weeks	F	*p*-value^c^	Difference in change	95% CI for change	F	*p*-value^d^
Pulldown/shoulder press	CHWIT control	25.3 ± 3.7 26.0 ± 3.7	28.5 ± 4.8 *^,a^	30.7 ± 5.0 *^,a,b^	32.6 ± 5.2 *^,a,b^ 25.1 ± 5.2	46.4	<0.001	7.1	5.3 to 8.9	68.7	<0.001
Adductor/abductor	CHWIT control	31.7 ± 6.2 33.3 ± 5.0	35.3 ± 5.2 *^,a^	39.1 ± 6.4 *^,a,b^	41.9 ± 5.7 *^,a,b^ 32.8 ± 4.8	28.6	<0.001	9.2	6.8 to 11.6	69.1	<0.001
Chest press/rowing	CHWIT control	22.6 ± 5.2 23.3 ± 5.3	25.0 ± 5.1 *^,a^	29.1 ± 6.1 *^,a,b^	31.3 ± 5.9 *^,a,b^ 24.6 ± 5.2	40.9	<0.001	6.6	4.3 to 8.9	42.1	<0.001
Hip extension 1	CHWIT control	27.4 ± 6.6 29.9 ± 5.7	33.1 ± 6.4 *^a^	35.0 ± 6.8 *^,ab^	38.3 ± 5.7 *^a,b^ 31.4 ± 5.8	44.2	<0.001	6.9	4.7 to 9.2	55.3	<0.001
Hip extension 2	CHWIT control	26.7 ± 5.5 30.3 ± 5.0	32.2 ± 6.4 *^,a^	34.7 ± 5.8 *^a,b^	37.9 ± 5.5 *^ab^ 29.8 ± 4.9	52.3	<0.001	8.0	5.6 to 10.4	60.8	<0.001
Back extension/flexion	CHWIT control	15.8 ± 4.0 16.0 ± 6.0	18.3 ± 4.6 *^,a^	20.3 ± 4.8 *^a,b^	22.3 ± 4.9 *^,a,b^ 15.5 ± 4.0	33.8	<0.001	6.8	4.8 to 8.7	50.1	<0.001
Butterfly	CHWIT control	21.2 ± 4.7 23.4 ± 4.2	25.3 ± 5.0 *^,a^	28.5 ± 5.3 *^a,b^	30.3 ± 4.6 *^,a,b^ 21.5 ± 4.3	46.4	<0.001	8.8	6.9 to 10.8	98.2	<0.001
Squat	CHWIT control	22.3 ± 5.6 22.9 ± 5.5	26.2 ± 5.9 *^,a^	27.9 ± 5.4 *^,a,b^	30.3 ± 5.5 *^,a,b^ 23.6 ± 5.0	20.8	<0.001	6.7	4.1 to 9.3	29.7	<0.001

The values were presented as mean ± standard deviation, mean difference in the change (post intervention value – pre intervention value), and 95% confidence interval (CI) for differences; CHWIT, circuit hydraulic weight-interval training-experimental group (n = 25); control, control group (n = 19); * statistically significant; ^a^ significant difference from baseline, *p* < 0.05; ^b^ significant difference from last measurement, *p* < 0.05; ^c^ calculated by multivariate tests for repeated measures ANOVA; ^d^ comparing between CHWIT and control group calculated by ANCOVA; analysis adjusted for baseline values.

## 4 Discussion

The aim of this study was to evaluate the influence of the CHWIT program, which combines hydraulic resistance with circuit and interval training, on the changes of anthropometric traits and body composition in pre- and postmenopausal women having a sedentary lifestyle. Menopause diminishes physical fitness and alters body composition by increasing total body fat and abdominal fat while decreasing fat-free mass, the main component of which is muscle mass. The latter is of key importance for maintaining independence in daily activities during the aging process progression. Muscle mass, especially their strength, as the studies of Newman et al. (2006) proved, is an important indicator of the muscle quality at the risk of mortality assessment. Regular exercise may reverse sarcopenia, prolong life, and improve its quality (Fragala et al., 2019).

The results of the current study confirm literature reports on the effects of periodic WT training on body composition components of the participants. A multitude of training systems offered to women by fitness clubs stimulates the search for training solutions that would bring positive health results while at the same time being safe and pleasurable for non-athletes. It was proved that WT, as a single form, or combined with other activities, twice or three times per week, is a basic strategy for the prevention and treatment of sarcopenia and its consequences (Hunter et al., 2004; Shaw et al., 2016; Foster and Armstrong, 2018). Standard strength training equipment, however, is mostly preferred by men whose main training aim is building the mass and strength of muscles. An alternative way is resistance training on hydraulic gym equipment, which does not expose the training person to risk of injury in the case of failure (Kerr and Comstock, 2010), and due to this, it is more willingly chosen by women and older people. A 12-week-long HWT protocol completed by women aged 65+ resulted in the improvement of body mass, fat-free mass, and muscle strength in upper and lower limbs (Gambassi et al., 2016). Similarly, older people (≥65 years) showed increased lower extremity muscle function after 10 weeks of HWT, with no effect on body weight (Dolan et al., 2015). In another study among older women (over 65 years of age) with sarcopenia obesity, a 10-week-long WT program proved not to be effective in enhancing their physical fitness (Vasconcelos et al., 2016). Only 24 weeks of WT, performed three times per week, in a group of women aged 60+ induced a significant growth of fat-free mass with no impact on the body fat mass (Gadelha et al., 2016). Positive effects on enhancing functional capability in women are obtained by combining resistance and circuit (CWT) or interval training (IWT). In young women, high-intensity circuit training (HICT) resulted in a significant decrease in total adipose tissue and subcutaneous leg and trunk fat (Trapp et al., 2008). In middle-aged and older women, CT for 12 weeks significantly increased fat-free mass, improved basic metabolism, and reduced BMI and adipose tissue (Hsu et al., 2015). Similarly, 10-month-long HIIT in women aged 36.4 (±4.4) years generated both muscle strength growth and caused a long-lasting negative energy balance and the permanent reduction of body mass and adipose tissue (Batrakoulis et al., 2018).

In the present study, only 6 weeks of CHWIT were enough to have a positive influence on reducing body fat in both groups of pre- and postmenopausal women. The changes in the muscle component were observable after 6 weeks in the premenopausal group, while in the case of postmenopausal women, another 12 weeks of training were necessary. The decrease in the size and number of muscle fibers accelerates considerably beyond 50 years of age, resulting in the loss of 30%–40% of muscle mass. A delayed reaction of muscle tissue to the training stimulus may result due to progression with age, diminished activity of satellite cells, age-related muscle hypertrophy limitation of an older muscle compared with a younger muscle as a result of a motor unit loss, and increased ingression of adipose and connective tissue into the muscles (Hunter et al., 2004). The research by Lehnert et al. (2015) showed that the body composition of inactive, premenopausal women aged 36.2 (±9.97) years after 12 weeks of CHWT did not change considerably. Kerksick et al. (2009) carried out a study among sedentary, obese, premenopausal women (38.5 ± 8.5 years) subjected to a 14-week CHWT program and suggested that the verification of the training effect shall be longer or supplemented with a nutrition plan in order to alter the body composition effectively. Similar conclusions were reached by Oliveira-Junior et al. (2021), who demonstrated improved aerobic capacity and flexibility without changes in body composition after 12 weeks of CWIT in adults without dietary recommendations or nutritional control. It should be noted, however, that a radical decrease in energy intake results in a reduction of lean body mass and has a negative effect on metabolism (Stiegler and Cunliffe, 2006). It was proven that in the case of older persons, the volume, frequency, ratio of workout to rest, and training intensity play a crucial role in the adaptations caused by CWT (Romero-Arenas et al., 2013). In the research carried out by Young (2007) involving postmenopausal women aged 56.5 (±7.5) years, 12 weeks of CHWT significantly reduced body mass and BMI but did not affect the muscle mass. In another study of women from 45 to 75 years of age, 12 weeks of CT caused a considerable decrease in body mass and adipose tissue and the increase in basic metabolism and lean body mass inclusive of muscle mass (Hsu et al., 2015). This study, however, did not take into consideration the status of menopause.

In the present study, adding elements of IT to the training of the investigated group of women allowed to achieve beneficial changes of body composition in a shorter period of time while maintaining the current nutrition model. Moreover, the training was of a changeable intensity, which is based on an alternate pace of resistance exercise and active rest. Lowering the body fat level, as observed in our study, may result from the introduction of short periods of active rest between the exercises, which intensifies the aerobic element of the program (Lee et al., 2011; Dupuit et al., 2021). At a training load of 60%–70% of maximum heart rate after 6 weeks of CHWIT, we noticed a statistically significant decrease in abdominal fat measured by waist circumference and WHR, although ANCOVA did not reveal any significant effects between groups (*p* > 0.05) (Table 1). Reduction of central fat has a significant impact due to the complications that are caused by such fat distribution in peri-menopausal women. A consequence of abdominal obesity is an increased incidence of coronary heart disease, diabetes, hypertension, and diminished quality of life (Teede et al., 2010). Increased efficiency of the circulatory system after 9 weeks of HWT was indicated by Cooney and Walker (1986). The changes in abdominal fat were observed after the trainings of high intensity. HIIT, promoted as a time-efficient strategy for improving body composition, significantly decreased abdominal and visceral fat mass in the sample of persons aged 38.8 (±14.4) years (Maillard et al., 2018), whereas 12-week-long HICT reduced waist circumference in persons aged 50 to 65 years (Paoli et al., 2010). In the research carried out by Maillard et al. (2018), HIT (over 90% of the maximum heart rate) was more effective in decreasing whole-body obesity, while lower rates of intensity had a higher impact on the changes in visceral and abdominal cavity fat mass. HIIT and moderate-intensity continuous training (MICT) in overweight or obese adults aged 18–45 years lead to a significant decrease in body mass, waist circumference, and whole-body fat (Wewege et al., 2017). Promising effects on improving various parameters of physical, mental, and metabolic health in inactive, overweight, and obese women are provided by new HIIT-type hybrid neuromuscular training protocols (Batrakoulis et al., 2020a; Batrakoulis et al., 2021b). The several-month hybrid training protocol proposed in these papers had positive effects on the body weight, abdominal obesity indices, resting cardiovascular function, glucose metabolism, lipid metabolism, and redox status (Batrakoulis et al., 2020a; Batrakoulis et al., 2021b); had improvements in mental wellbeing and subjective vitality (Batrakoulis et al., 2020a); and improved musculoskeletal fitness indicators (Batrakoulis et al., 2020b; Batrakoulis et al., 2023).

The results of the present study prove that a low level of resistance is enough to activate the growth of muscle strength. Other studies have shown that WT programs with low or moderate load (45% 1RM) induce strength and skeletal muscle hypertrophy in persons over 65 years of age under the condition that a sufficient number of repetitions is performed (Csapo and Alegre, 2016). Owing to that, HWT is recommended as an effective form of resistance training that provides benefits for older adults even at low or moderate intensity of training (Lee et al., 2011). In the exercises on hydraulic equipment, the level of resistance intensity is determined by the individual effort and workout speed of the participant at each repetition (Takeshima et al., 2002). In the present study, a gradual increase in the number of repetitions on the hydraulic exercise machines was observed in both age groups at every stage of CHWIT. Therefore, the training scheme proposed in this study is useful to initiate changes affecting muscle mass conditioning.

It is important to emphasize the effect of HWT (adductor/abductor hydraulic machine; Tables 3, 4) on the function of the hip adductor and abductor muscles. These muscles are responsible for proper hip function, which, in the elderly female population, is one of the most common sites of injury that can lead to falls (Gafner et al., 2021). Hydraulic resistance machines are designed to exercise both muscle groups so that the muscle force is evenly distributed, preventing hip overload and injury. Such a system is suitable for functional rehabilitation, improving the range of motion, muscle strength, symmetry of movement, and motor coordination. Notably, in the study group of women, we did not note any report of injury occurrence during the training program. Therefore, HWT is a safe form of training in the elderly female population that affects the muscular balance of the hip joint. Comparative studies of different populations (athletes and non-exercisers, young and elderly persons) show that the pattern of improvement in hip adductor and abductor muscle strength can differ and depends on many factors (Kemp et al., 2013; Gafner et al., 2017; Karatrantou et al., 2019; Jaenada-Carrilero et al., 2024). Nadler et al. (2000) showed that the coefficient of variation for hip abduction strength in young individuals was 26%, while in the elderly population, it was 49% (Gafner et al., 2017), which makes this trait clinically relevant and important in physiotherapy procedures (Kemp et al., 2013). Research is currently underway to refine and standardize methodologies for measuring hip adduction and abduction strength in aging populations, which will enable repeatability of investigations and comparison of results (Gafner et al., 2017; Gafner et al., 2021; Kawakami et al., 2024).

Our findings support the conclusions that a hybrid-type exercise approach integrating endurance exercise and alternative resistance-based modes improves body composition parameters and musculoskeletal fitness in overweight and obese women with sedentary lifestyles (Batrakoulis et al., 2020b). Such protocols provide knowledge of beneficial health adaptations achievable during short, supervised training sessions, which sets the stage for future research based on longitudinal interventions in the real-world (Batrakoulis et al., 2019).

The strength of this study is its long-term follow-up and comparative analysis of the effects of circuit hydraulic weight interval training on body composition parameters in pre- and postmenopausal women. In addition, the participants’ exercise performance was fully supervised by the trainer throughout the study.

However, there were some unavoidable research limitations in this study. Due to the length of the experiment and its implementation in a recreational sports center, there were difficulties in maintaining a random selection of participants in the groups. Therefore, a quasi-experimental study was used, conducted in two age groups, whose primary limitation was the lack of randomization. In addition, selection bias may have occurred. Women who participated in the intervention study and attended the exercise sessions may have had stronger health-promoting motivation compared with women who entered the study through open recruitment but attended only the baseline and end-line measurements.

Another limiting factor in this study may be the lack of nutritional control. Our participants only declared no change in eating habits. We would like to point out that it is difficult to keep more people on the 18-week training program (54 training sessions) with strict nutritional control at the same time. We also wanted to avoid underestimating the calorie intake of the study participants, which can happen in reports based on 24-hour dietary recalls (Mitka, 2013).

We recognize that a limitation to our study may be the use of BIA to assess body composition parameters, especially as DXA is considered the reference method. A recent retrospective study comparing DXA and BIA measurements of nutrition department patients showed that both methods can be used interchangeably to assess body composition at the population level (Achamrah et al., 2018). Similarly, other studies have shown that the BIA method can be a viable alternative to DXA in different groups of men and women, including physical training persons and patients (Anderson et al., 2012; Benito et al., 2019; Antonio et al., 2019; Cruz Rivera et al., 2022). We assure that, in accordance with the BIA measurement methodology, every effort was made to maintain proper body hydration in the study participants. BIA is a safe, practical, and reliable method of assessing body composition with increasing use in sports (Dżygadło et al., 2012), and in the case of field investigations and in gyms and fitness clubs, it is the only one available due to the cost and location of the study. Participants in such surveys praise the short measurement time and immediate assessment of body composition components, which can positively motivate people to exercise. When maintaining the repeatability of the test conditions, long-term comparative observations can be made at the training site.

Finally, we did not conduct an observation to determine the long-term effects of participation in this type of exercise intervention. Therefore, further research is also needed to assess the feasibility of maintaining the usefulness and effectiveness of CHWIT on body composition and muscle mass condition in peri-menopausal women. In this study, we were more interested in determining the direction of change and the period of time needed to remodel the body composition of the studied groups of women under the influence of CHWIT. At the present time, our study is preliminary and more exploratory than conclusive.

## 5 Conclusion

Despite the study’s limitations, the results suggest that the CHWIT protocol, which is part of Mrs.Sporty’s commercial program, using hydraulic resistance, circuit exercise, and interval training, may be recommended for pre- and postmenopausal women to reduce body fat and improve anthropometric characteristics, body composition, and physical fitness. Significant changes in the muscle component estimated by BIA require a longer exercise intervention in women after menopause. Fitness specialists may advise peri-menopausal women CHWIT exercises, with breaks on the active rest stations, to ensure healthy and independent aging.

## Data Availability

The original contributions presented in the study are included in the article/supplementary material; further inquiries can be directed to the corresponding author.
